# Kindlins in the cardiovascular system: from development to pathogenesis

**DOI:** 10.3389/fcvm.2026.1795930

**Published:** 2026-04-01

**Authors:** Qi Wang, Yuechao Dong

**Affiliations:** 1 Department of Clinical Laboratory Medicine, Jiamusi Infectious Disease Hospital, Jiamusi, China; 2 Department of Clinical Pharmacology, College of Pharmacy, Harbin Medical University, Harbin, China

**Keywords:** cardiovascular, ECS, integrin, kindlin-2, kindlin-3

## Abstract

Over a decade ago, Kindlin-2 was first identified as an important effector in heart development. Thereafter, a series of studies demonstrated the pivotal roles of Kindlins in the cardiovascular system. The integrin-dependent and -independent downstream signals of Kindlin-2 is now widely recognized to be essential in many events in cardiovascular system including angiogenesis, vascular permeability, cardiac development. Furthermore, the important role of Kindlins has been demonstrated in the pathogenesis of arteriosclerosis, ischemia/reperfusion injury, acute lung injury, pulmonary hypertension, heart failure and even osteoporosis. Collectively, Kindlins serve as crucial mediators under both physiological and pathological conditions and represent promising therapeutic target in cardiovascular medicine.

## Introduction

Focal adhesions (FAs), the integrin-containing complexes linking cytoskeleton to extracellular matrix (ECM). Dynamically assembled and disassembled of FAs are orchestrated upon a spatiotemporal manner (1). A sophisticated network of protein-protein interactions within FAs precisely orchestrates fundamental cellular processes such as adhesion, spreading, migration, and survival. Kindlins are the key components of FAs. Accelerating research identified kindlins recruited paxillin to activate focal adhesion kinase (FAK) (2). Kindlins also closely correlated with integrin-linked kinase (ILK) to form complex with pinch and parvin (3). Consequently, Kindlin and the associated proteins in nascent FAs are responsible for FA maturation through facilitating actin cytoskeleton remodeling (4).

To date, three members of the Kindlin family (Gene name is Fermt) have been discovered. Kindlin-1 is primarily found in ectodermal cells, kindlin-3 is majorly restricted to hematopoietic cells. Kindlin-2 is extensively distributed across various cells (5). Mutations of FERMT1 gene located on chromosome 20p12.3 induced Kindler syndrome, a rare autosomal recessive genodermatosis characterized by fragile skin, photosensitivity, skin heterochromia, and mucosal damage, etc. Mutations in the FERMT3 gene located on chromosome 11q13.1 induced Leukocyte Adhesion Deficiency-III with symptoms of recurrent infections, poor wound healing, and a pronounced tendency to bleed. Nowadays, there is no syndrome induced by Fermt2 mutation (6, 7).

Cardiovascular system is particularly rich in kindlin-2. Endothelial cells (ECs) and cardiac fibroblasts uniquely co-express Kindlin-2 and kindlin-3. However, only Kindlin-2 was discovered in cardiomyocytes. Kindlins contain a conserved FERM domain (F1-F3), shared with protein 4.1, ezrin, radixin and moesin (abbreviations of the 4 first letters above is FERM) (8). Being characterized by insertion of PH domain in F2, Kindlins can localize on membrane and activate integrins through interacting with cytoplasmic tails (9). Integrins on the cell membrane coordinate cellular behaviors precisely through bidirectional signaling: “inside-out” and “outside-in” (10, 11). Integrin dysfunction contributes to cardiovascular diseases such as atherosclerosis, hypertension, thymbrosis, cardiac fibrosis, and heart failure, etc. (12–15). The integral involvement of kindlins in integrin activation has been collected from different investigating lines.

In addition to integrin activation, Kindlins also bridge the linkage between functional proteins to affect downstream pathways. Generally, post-translational modification, such as ubiquitination, phsphorylation and methylation etc., occurs to affect the stability of target proteins. Therefore, we will review the current research highlighting the multifaceted significance of kindlins in the cardiovascular system (Table 1).

**Table 1:** Multifaceted significance of kindlins in the cardiovascular system.

Physiological/Pathological events	Cell type and Kindlin member	Mechanism	Function
Heart development	Kindlin-2 in cardiomyocytes	-	Kindlin-2 KO results in cardiac hypoplasia (16).
integrity of the Z-disc	Kindlin-2 in cardiomyocytes	Kindlin-2/integrinβ1/α-actinin form a complex	Kindlin-2 KD led to the disrupted sarcomere (18).
Progressive heart failure	Kindlin-2 in cardiomyocytes	Stablize integrin β1D	Kindlin-2 deficiency induce Progressive heart failure (17).
Cardiac hypertrophy	Kindlin-2 in cardiomyocytes	Recruit SUV39H1 to mediate histone methylation of GATA promoter	Kindlin-2 inhibits cardiac hypertrophy (20).
Myocardial infarction	Kindlin-3 in ECs	Reinforce β1-integrin to activate Notch	Endothelial Kindlin-3 improves Myocardial infarction (23).
Cardiac ischemic reperfusion	Kindlin-2 in cardiomyocytes	Enhance Otub1/Slc7a11	Kindlin-2 inhibits Cardiac ischemic reperfusion (21).
Cardiac fibroblast activation	Kindlin-2 in fibroblasts	Activate α-SMA promoter	Kindlin-2 mediates TGF-β/stiff ECM-activated fibroblast (25).
Cardiac fibroblast activation	Kindlin-2 in fibroblast	Inhibit ERK phosphorylation	Kindlin-2 deficiency decreased actin polymerization (26).
angiogenesis	Kindlin-2 in ECs	Activate Integrin αVβ3	Kindlin-2 deficiency induces impaired angiogenesis and leaky vasculature (27).
angiogenesis	Kindlin-2 in ECs	Disrupt Notch1 pathway	Kindlin-2 promotes physiological angiogenesis (5).
angiogenesis	Kindlin-2 in ECs	Induces nucleus transforming of β-catenin	Endothelial Kindlin-2 accelerates the wound healing (31).
Vascular integrity	Kindlin-2 in ECs	Strengthen VE-cadherin/catenin complexes to stabilize vascular barrier.	Kindlin-2 KO aggravates lipopolysaccharide-stimulated vascular leakage and pulmonary oedema (33).
Atherosclerosis	Kindlin-2 in ECs	Arginine methylation of Kindlin-2 impair FA assembly and junction maturation.	Endothelial Kindlin-2 KO Mice presented increased atherosclerosis lesions (4).
Acute lung injury	Kindlin-2 in ECs	Interact with integrin β4	Kindlin-2 alleviates inflammatory ECs of ALI (34).
Vascular integrity	Kindlin-2 in mural cells	Interact with integrins	Kindlin-2 in mural cells promoted the adhesion, contractile and survival of vSMCs and pericytes to stabilize vessels (35).
leukocyte adhesion deficiency III	Kindlin-3 in leukocytes	Interact with β2 integrin	Kindlin-3 KO disrupted adhesion of leukocytes on ECs and severe hemorrhage (36).
platelet aggregation	Kindlin-2 in platelet	Kindlin-2 interact with clathrin to mediates endocytosis of CD39 and CD73.	Kindlin-2 triggers platelet aggregation (39).
Osteoporosis	Kindlin-2 in ECs	Induce deubiquitination of Piezo1 to activate TGFβ/RUNX2 in osteoblasts	Eneothelail Kindlin-2 Inhibit osteoporosis (40).

## Kindlin-2 in heart development and homeostasis

Global kindlin-2 mutation results in embryonic lethality at E7.5. As the first developed organ during embryogenesis, the heart undergoes the process from embryonic development to maturity. CM-specific Kindlin-2 KO occurs in embryonic mice (E8.0) and results in embryonic lethality. Therefore, no Kindlin-2^f/f^-cre mice was acquired after birth. Subsequently, evident Kindlin-2 decreasing at P14 mice led to premature death (16, 17). Altogether, Kindlin-2 is essential in cardiac development from embryonic stage to adulthood. Molecular mechanisms orchestrating cardiogenesis from scratch are responsible for maintaining the integral form and function.

In 2008, Kindlin-2 is firstly detected in hearts and enriched in intercalated discs, which is crucial for cytoskeletal organization at sites of membrane attachment to maintain cardiogenesis and functions. Kindlin-2 mutation results in cardiac hypoplasia with tinn walls, dilated chambers and abnormal contractility (16). Next, Kindlin-2/integrin β1 was identified to form a complex with actinin-2, an important structural component of the Z-disc. Kindlin-2 KD led to the fragmented Z-disc and disrupted sarcomere. Therefore, Kindlin-2 ensures the integrity of the Z-disc (18). And in cardiac muscles, depletion of Kindlin-2 in mice leads to the disordered myocardial fibers, systolic functions, and even the cardiac hypertrophic cardiomyopathy (19). These cardiac dysfunctions resulting from Kindlin-2 deficiency promote further research of Kindlins' involvement in cardiopathy.

## Key events related to effects of kindlins on heart diseases

Kindlins are the significant factors sustaining the integrity of cardiovascular system. Kindlins deficiency heralds the occurrence of various cardiovascular events such as atherosclerosis, acute lung injury (ALI), pathological hypertrophy cardiomyopathy, etc. Therefore, the protective effects of Kindlins on cardiovascular diseases are summarized in the following context.

### Kindlins in postnatal deletion and heart failure phenotype

Lacking Kindlin-2 in early developing heart results in embryonic lethality. Deletion of Kindlin-2 at late gestation or in adult cardiac myocytes resulted in heart failure and premature death, which was associated with enlargement of the heart, and extensive fibrosis. In addition, integrin β1D protein expression was significantly downregulated in adult heart. Kindlin-2 is required to maintain integrin β1D protein stability. Postnatal loss of Kindlin-2 from cardiomyocytes leads to progressive heart failure showing the importance of costameric proteins like Kindlin-2 for homeostasis of normal heart function (17).

### Kindlins in hypertrophy signaling

Prevention effects of Kindlin-2 in cardiopathy was confirmed in 2019. Qi et al. discovered that Kindlin-2 suppresses the expression of GATA4 through binding to its promoter and prevents cardiomyocytes from hypertrophy induced by isoproterenol (ISO) treatment. As a member of the GATA transcription factor family, GATA4 activates transcription of hypertrophic responsive genes. Kindlin-2 interacts with histone methyltransferase SUV39H1 and recruits it to GATA4 promoter leading to the occupancy of histone H3K9 di- and tri-methylation. On the contrary, depletion of Kindlin-2 contributes to cardiac hypertrophy and progressive heart failure. GATA4 expression was markedly activated in cardiac tissues of Kindlin-2 cKO mice compared to wild-type group (20).

### Kindlins in myocardial ischemia

Ischemic heart disease is caused by coronary artery stenosis or plaque rupture, resulting in reduced myocardial blood flow or oxygen supply. Reperfusion aiming to control the ischemia injury can deteriorate cardiac trauma, which is termed as cardiac ischemia reperfusion (I/R) injury and cardiomyocyte ferroptosis is regarded as the important contributory mechanism (21). Kindlin-2 level in the peri-infarct region of I/R heart tissues decreased in a time-dependent manner. While it remains unaltered in the infarct distal zone of tissue specimens. Therefore, this suggested that Kindlin-2 is potential to improve ischemia cardiomyopathy. As an ovarian tumor (OTU) family member deubiquitinase, Otub1 stabilizes Slc7a11 and its downstream GSH/GPX4 axis to prevent ferroptosis. Through enhancing the interaction between Otub1 and Slc7a11, Kindlin-2 contributed to the mitigate cardiac I/R injury through inhibiting ferroptosis (22).

Moreover, therapeutic angiogenesis, which improves blood flow recovery after ischemia injury, has been extensively regarded as the promising strategy to restore heart failure post-MI. Kindlin-3 in cardiac microvascular endothelial cells (CMECs) promotes angiogenesis through reinforcing β1-integrin to activating Notch pathway. Consequently, MI-induced cardiac dysfunction, cardiomyocyte apoptosis and myocardial fibrosis was alleviated (23).

Previous studies also have revealed the interaction between Kindlin-2 and HIF-1α in cardiomyocytes and breast cancer cells (MCF) (22, 24). Invasive breast cancer can be accelerated by abundant Kindlin-2 and HIF-1α. In contrast, HIF-1a and Kindlin-2 in H/R-stimulated cardiomyocytes were decreased. Furthermore, as a transcriptional factor, HIF-1a positively regulates Kindlin-2 in MCF. HIF-1α promotes angiogenesis and vascular remodeling by modulating VEGF, which is acknowledged as the most specific pro-angiogenic cytokine. Therefore, the involvement of Kindlin-2 in HIF-1a-induced collateral circulation in myocardial infarct tissues should be explored in the future research.

### Kindlins in myocardial fibrosis

In response to pathological events, cardiac fibroblasts sense and alter the properties of ECM to make the heart adapt to mechanical stress or improve lesions. Under the aberrant mechanosensing, cardiac fibroblast differentiate into myofibroblasts which secrete more collagens. Consequently, the scar tissues composed of excessive collagen is termed as myocardial fibrosis. As the key molecule of mechanotransduction pathway, kindlin-2 are elevated in TGF-β1 or stiff matrix-cultured cardiac fibroblasts and fibrotic hearts. In stressing fibroblasts, kindlin-2 translocate to the nucleus to activate α-SMA promoter through CC(A/T)-rich GG(CArG) box elements (25). In hearts of aging male mice with stiff ECM, decreased actin polymerization of fibroblasts results from Kindlin-2 deficiency-induced ERK phosphorylation. The old-like fibroblasts can be reversed by Kindlin-2 OE. In aging female mice, cardiac fibroblasts remains young-like by the advantage of unaltered Kindlin-2 compared with the young group. Consequently, high level Kindlin-2 can bypass the ECM components to favor the differentiation of fibroblasts into myofibroblasts. It demonstrates differences responses of cardiac fibroblasts between male and female to stiff ECM (26).

## Kindlins in EC dysfunction

### Kindlins and angiogenesis

Physiological angiogenesis under the balance of pro and anti-angiogenic factors is crucial for development. In contrast, pathological angiogenesis caused by the disrupted balance manifests as excessive, thinner and more permeable vasculature and aggravates various diseases. The indispensability of Kindlin-2 in developmental angiogenesis was primarily verified in Zebrafish. And ECs derived from kindlin-2 deficiency mice have disrupted αVβ3-dependent signaling, impaired angiogenesis and leaky vasculature (27). Moreover, both developmental and tumor angiogenesis rely on the outside-in signaling of ECs, which is promoted by the interaction of the integrin αVβ3 cytoplasmic tail with kindlin-2 (28). Kindlin-2 deficiency-induced leaky vasculature also prevented wound healing of skin (29). In contrast, kindlin-2 overexpression also contributes to the transforming of β-catenin from cytoplasm to nucleus. In view of promoting the division and chemotaxis of ECs and enhancing VEGF expression, activated Wnt/β-catenin signaling accelerates the wound healing through affecting vascularization (30). Although Kindlin-2 is implicated as an angiogenic promoter, the endothelium-specific Kindlin-2 in regulating angiogenesis remains further demonstration. Angiogenesis requires the cooperation between tip cells and stalk cells, the two adjacent with opposite function ECs are orchestrated by VEGF/Notch pathway. Activated Notch pathway, characterized by release of Notch intracellular domain, in ECs contributes to stalk cell phenotype. By binding to and maintaining the integrity of NOTCH1, endothelial Kindlin-2 prevents Notch pathway to induce sprouting angiogenesis (5). In addition to the physiological angiogenesis, elevated kindlin-2 in tumor also promotes tumour angiogenesis through the mTOR/VEGFA pathway in melanoma (31). Endothelial Kindlin-2 deficiency also prevent high glucose-induced hyperactive angiogenesis *in vitro* (5).

Although being 20%–50% of Kindlin-2 in endothelial cells, Kindlin-3 was also verified to promote angiogenesis through activating integrin β3-Twist crosstalk. As a transcriptional factor, nuclear localization of Twist is known to promote tumor angiogenesis through producing VEGF (32). It is worth noting that exogenous VEGF increases endothelial Kindlin-2, which also enhances the level of Kindlin-3 without certain mechanism (5). Taken together, Kindlin-2 and Kindlin-3 are potential to generate the positive feedback loop during angiogenesis. Therefore, Kindlins are highlighted as the potential target in angiogenic events, such as tumor, diabetic retinopathy, and wound healing etc.

### Kindlins in vascular permeability

The adherens junctions (AJs) are particularly crucial for maintaining vascular integrity and required for proper assembly of tight junctions. Endothelial AJs are formed by VE-cadherin, which binds to intracellular partners (α-catenin, β-catenin, γ-catenin) to interacts with actin cytoskeleton. Stable and mature AJs contribute to quiescent endothelium and vascular integrity.

Kindlin-2 stabilizes AJs through functioning on β- and γ-catenin within the F1 and F3 subdomains. The strengthened VE-cadherin/catenin complexes was linked to cortical actin to stabilize AJ and maintain the vascular barrier. Kindlin-2 and cortical actin can dissociate from AJs and generate radial actin stress fibres when ECs are stimulated by thrombin *in vitro*. Lipopolysaccharide-stimulated vascular leakage and pulmonary oedema can be aggravated by Kindlin-2 deficiency (33).

Acute lung injury (ALI) lacks of effective treatment and mitigating the permeability of ECs in lungs is gradually recognized as the strategies of ALI. Impairment of barriers between blood and surrounding tissues, established by endothelium, prevailingly occurs during inflammation and causes severe dysfunction of affected organs. As an HMG-CoA reductase inhibitor, Simvastatin enhances the association of Kindlin-2 and integrin β4 to alleviate inflammatory ECs of acute lung injury (ALI). Kindlin-2 prevents Thrombin-induced EC barrier disruption and Kindlin-2 deficiency induces human lung EC barrier dysfunction (34). In addition to ECs, Kindlin-2/integrin promoted the adhesion, contractile and survival of vSMCs and pericytes to cover endothelium and stabilize vessels. In contrast, reduced integrin activation derived from Kindlin-2 KO in vSMCs and pericytes results in the detachment of the mural cells from vasculature, vascular leakage (35).

### Kindlins in vascular inflammation

Kindlin-3/Integrin activation is indispensable for the adhesive function of platelets and leukocytes. Kindlin-3 mutated humans lose the activation of β integrins on platelets and leukocytes termed as leukocyte adhesion deficiency III (LAD-III), a rare syndrome characterized by disrupted adhesion of leukocytes and severe hemorrhage. Neutrophil Kindlin-3 is essential for binding the ligands of β2 integrin. The firm arrest of neutrophils on endothelium was abolished by Kindlin-3 deficiency and mice phenotype of Kindlin-3 mutation is LAD-III like (36). As the focal adhesion protein, Kindlin-2 and Kindlin-3 mediate ECs adhesion upon the combination of integrins and substrates. However, the integrin and substrate controlled by Kindlin-3 is distinct from that of kindlin-2. Endothelial Kindlin-3/integrin α5β1 mediate the cell adhesion to fibronectin. Endothelial Kindlin-2/integrin αvβ3 mediate the cell adhesion to vitronectin. Kindlin-3 deficiency impaired adhesive functions despite the presence of kindlin-2 (37). Combined the results of the two publications, leukocyte-ECs interactions depends on Kindlin-3 in leukocyte rather than ECs. As expected, Kindlin-3 was also discovered to be rich in the monocytic origin cells and M2 type macrophages within the unstable plauques of atherosclerosis patients (38).

Being in an integrin-independent manner, interaction of kindlin-2 F3 subdomain with clathrin heavy chain mediates endocytosis of adenosine triphosphate (ATP) - diphosphate hydrolase (CD39) and ecto-5'-nucleotidase (CD73) to trigger platelet aggregation. Kindlin-2 deficiency leads to the degradation of adenosine 5'- diphosphate (ADP) by enhancing the trafficking of CD39 and CD73 at the cell surface. Enhanced ATP/ADP catabolism and production of adenosine inhibits platelet aggregation (39).

Unidirectional blood flow in descending thoracic aorta (TA) is atheroprotective. Endothelial Kindlin-2 localized at the borders of ECs preserves barrier function of endothelium. Atheroprone shear stress in the inner curvature of the aortic arch (AA inner) is low and oscillatory. Endothelial Kindlin-2 showed a predominantly cytoplasmic localization enhances endothelial permeability. Endothelial Kindlin-2 KO Mice presented increased vascular permeability and atherosclerosis lesions. Mechanistically, PRMT5 induce the arginine methylation of Kindlin-2 at R290 to impair FA assembly and junction maturation. Although ECs rich in Kindlins are more adhesive, endothelial Kindlins do not primarily mediate leukocyte-ECs interactions during inflammation. Therefore, targeting Kindlin-2 arginine methylation emerges as a promising hemodynamic-based strategy for treating vascular disorders and atherosclerosis (4).

## Related role: endothelial kindlin-2 in osteoporosis

In bone tissues, endothelial Kindlin-2 deficiency causes the deubiquitination of Piezo-1, the calcium channel senses fluid shear stress, to increase the osteogenesis through activating TGFβ/RUNX2. RUNX2 is the runt-related transcription factor controlling osteoblast differentiation. In contrast, increased endothelial Kindlin-2 are observed both in mice and humans with osteoporosis. Therefore, Kindlin-2/Piezo1/TGFβ/Runx2 axis is potential to be the promising therapeutic strategy for OP (40).

## Conclusion and perspective

**Figure 1:**
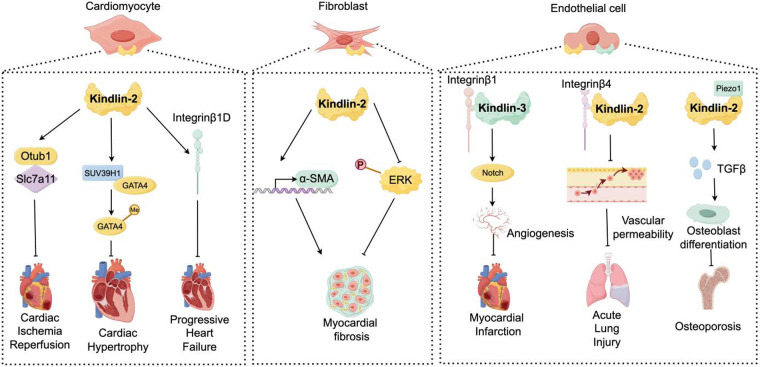
Kindlins in cardiovascular system improve various diseases.

Collectively, a comprehensive description of Kindlins in cardiovascular system has been systematically outlined. Through regulating integrin-dependent and -independent pathways, Kindlins are essential for sustaining cardiac functions and vascular integrity under pathological conditions (Figure 1). Despite significant advances, we proposed several challenges the research around Kindlins maybe face in the future:

1. The development of Kindlin-targeted therapies requires efficacy and safety. Small-molecule compounds or peptide-based strategies targeting Kindlins should be developed to minimize Off-target effects.
2. Discrepancy of Kindlin-2 in myocardial fibroblasts caused by age or sex may help to provide personalized therapeutic approaches for clinical heterogeneity of fibrotic diseases
3. Based on the existing results, future studies should validate the functional heterogeneity of Kindlins in pulmonary arterial hypertension, cardiac fibrosis, atherosclerosis and diabetic vasculopathy, etc. The landscape of Kindlins in cardiovascualr system should be further mapped. Validate whether Kindlins can serve as early diagnostic biomarkers or prognostic indicators for these diseases.
4. Post-translational modifications of Kindlins in response to hemodynamic or metabolic stress should be investigated. Kindlin stability, subcellular localization, and interactions with effector proteins in response to PTM reveal novel mechanisms in cardiovascular pathogenesis.

Kindlin-regulated signals are poised at the frontiers of cardiovascular biology and provided promising perspective for the innovative diagnostics and therapies of cardiovascular diseases.
